# “If You Haven’t Slept a Lot (…) You Don’t Want to Go Out for a Run, You Don’t Want to Ride a Bike, You Just Kind of Sit and You Just (…) Do Nothing”—Perceptions of 24-Hour Movement Behaviours Among Adolescents Living with Type 1 Diabetes

**DOI:** 10.3390/ijerph22081295

**Published:** 2025-08-19

**Authors:** Mhairi Patience, Alison Kirk, Xanne Janssen, James Sanders, Megan Crawford

**Affiliations:** 1 Psychology Group, Department of Psychological Sciences & Health, Faculty of Humanities & Social Sciences, University of Strathclyde, Glasgow G1 1XP, UK; 2 Physical Activity for Health Group, Department of Psychological Sciences & Health, Faculty of Humanities & Social Sciences, University of Strathclyde, Glasgow G1 1XP, UK; 3 School of Sport, Exercise and Health Sciences, Loughborough University, Loughborough LE11 3TU, UK

**Keywords:** adolescent, sleep, physical activity, sedentary behaviour, type 1 diabetes, qualitative, 24-hour movement behaviours

## Abstract

The importance of physical activity, sedentary behaviour, and sleep behaviour in adolescents with type 1 diabetes (T1D) has been explored in isolation. However, adolescents experience health benefits when these behaviours are balanced appropriately throughout the day, and are considered to be interconnected rather than isolated. The aim of this study was to investigate the perspectives of adolescents living with T1D towards these behaviours collectively. The participants were 15 adolescents (aged 11–18 years) with T1D, recruited using online methods and word of mouth. Online semi-structured interviews were transcribed using intelligent verbatim and analysed using thematic analysis. We identified the following four central themes and five subthemes: (1) sleep and physical activity are understood and valued above sedentary behaviour; (2) recognition of movement behaviours’ interconnection; (3) movement behaviours’ interaction with health outcomes (mood, glycaemic control, and glycaemic control as a barrier to movement behaviours); and (4) movement behaviours within the environmental context of the adolescent (school and caregivers). Adolescents with T1D are aware of the interconnectedness of each movement behaviour and the positive influence a balanced approach can have on mood and T1D management. The findings provide important information for future holistic interventions promoting healthy behaviours that target the adolescent, their school environment, and their caregivers.

## 1. Introduction

Type 1 diabetes (T1D) necessitates 24-hour management, involving careful and constant monitoring of blood glucose levels, insulin administration, and lifestyle adjustments [1]. This attention and adaptation creates unique challenges, particularly for adolescents, who form a significant demographic of those living with type 1 diabetes and already navigate a myriad of changes associated with adolescence (e.g., increased autonomy, expanded social interactions, and physiological transformations) [2,3]. Every aspect of their daily routine holds implications for glucose control and management decisions, including the triad of movement behaviours performed during a 24 hour day, namely, physical activity (PA), sedentary behaviour (SB), and sleep.

Research has consistently demonstrated the positive effects of increased physical activity and reduced sedentary behaviour on glucose control and psychosocial outcomes within the type 1 diabetes adolescent population, and they are recognised as a cornerstone of type 1 diabetes management. Guidelines recommend at least 60 min of moderate–vigorous intensity PA (MVPA) per day and to limit sedentary behaviour [4,5,6]. Although there are no sleep guidelines specific to this population, suboptimal sleep duration and poor sleep quality in this population have been recorded. A bi-directional relationship between sleep and glycaemic control has been evidenced by sleep disturbances related to glucose variability, diabetes distress, and the completion of important type 1 diabetes self-management activities in adolescence [7,8,9]. However, no research has investigated how combinations of all three behaviours interact and impact on important type 1 diabetes outcomes [10].

The 24-hour movement behaviour (24-h MB) approach argues for the combined examination of all movement behaviours [11]. Physical activity, sedentary behaviour, and sleep should be treated as part of a continuum from no movement [12] to high movement (vigorous physical activity). These behaviours are considered to be relative or time-dependent, meaning a change in one behaviour can impact another. Importantly, the way these movement behaviours are combined throughout the day has significant implications for the physical and mental health of adolescents [13,14]. This evidence has resulted in the development of 24-h MB adolescent guidelines and the incorporation of key 24-h MB principles in global movement behaviour guidelines, highlighting the detrimental effect of sedentary behaviour on sleep duration, the positive influence of physical activity on sleep, and the importance of replacing sedentary behaviour with physical activity [15,16].

The current qualitative research on adolescents with type 1 diabetes has investigated perceptions of sleep, sedentary behaviour, and physical activity individually; however, no study, to our knowledge, has investigated how adolescents with type 1 diabetes collectively understand these behaviours [9,17,18,19]. Given the increased attention towards a 24-h MB approach, the evidence indicating the influence of each behaviour on important type 1 diabetes outcomes, and the 24-hour management required for type 1 diabetes, a holistic—rather than isolated—approach towards movement behaviours should now be considered. A crucial step towards this is to initially investigate the current understanding of adolescents living with type 1 diabetes towards the interconnected nature of these behaviours and how they might impact health. This provides a starting point in the development of effective interventions aimed at supporting type 1 diabetes management in this population.

## 2. Materials and Methods

### 2.1. Study Design

This qualitative study was part of a larger piece of research exploring 24-h MBs in adolescents living with type 1 diabetes with both quantitative (questionnaire and wrist-worn accelerometer) and qualitative elements. Semi-structured interviews were conducted after the individual completed the quantitative part of the research project. The qualitative approach was informed by concepts of interpretivism, aiming to understand the experiences and perceptions of movement behaviours of adolescents with type 1 diabetes [20]. Additionally, pragmatism concepts were considered for this study to facilitate the practical implications of the research [21]. The University of Strathclyde Research Ethics Committee reviewed and approved this research (UEC22/04) on 27 April 2022.

### 2.2. Participants

Participants were eligible for this qualitative study if they were aged 11–18 years, were diagnosed with type 1 diabetes, currently lived in the UK, spoke English, had no mobility-related issues or required walking aids, and had not been diagnosed with a sleep disorder (self-reported). These exclusion criteria were applied to reduce potential confounding factors that could independently affect movement behaviours. Recruitment materials included infographics and a video of the lead researcher describing the research, aiming to engage the target group more successfully [22].

Participants were recruited using online methods (e.g., social media) and word of mouth. Diabetes charities and type 1 diabetes influencers were involved in the dissemination of recruitment materials throughout a six-month period between March 2023 and August 2023. Recruited participants completed a prior study involving accelerometer-based measurement of their movement behaviours. Although behavioural diversity was not used as a recruitment criterion, the sample reflected a range of movement profiles, including differences in physical activity, sedentary behaviour, and sleep duration, as observed in the accelerometer data. Not all participants provided valid movement behaviour data due to insufficient wear time. However, all who were enrolled completed the qualitative interview, and no participants were lost to follow-up.

Interested individuals were then directed to an initial questionnaire to assess eligibility and the appropriate consent pathway. Participants provided consent to conduct the study and publish the study. For adolescents aged 11–15 years (younger adolescents), written consent was obtained and a verbal consent meeting was conducted with the adolescent and their caregiver to gain verbal consent/assent. For adolescents aged 16–18 years (older adolescents), written consent was obtained.

### 2.3. Data Collection

Upon enrolment into the study, participants completed a questionnaire that assessed demographic information and diabetes-specific information (e.g., insulin delivery and blood glucose measurement methods, diabetes duration, and mean haemoglobin A_1c_ (HbA_1c_)). Health-related quality of life was measured using the validated Paediatric Quality of Life (PedsQL) 3.2 Diabetes Module for adolescents with type 1 diabetes (use of this questionnaire was approved by the author in December 2021) [23]. After returning an ActiGraph GT3X-BT accelerometer (please see Supplement S1 for accelerometer data processing methods), an interview was scheduled for those who agreed to take part in this qualitative part of the study.

Semi-structured interviews were arranged at a suitable time for participants and were conducted online via Zoom, allowing for audio and video recording [24]. All participants completed the interviews within their home environment and without a caregiver present to ensure the conversation was not influenced and communicated perceptions were original. Interviews were conducted by the lead researcher, a 28-year-old female currently completing her PhD, who had trained in video conferencing platform interview techniques and specialised in qualitative methods during their Master’s degree (M.P.; MSc). Rapport had previously been established between the interviewer and adolescent during the accelerometer data collection phase. Rapport is theorised to enhance trust and confidence, resulting in improved discussion [21]. The interview utilised an informal conversational style and was guided by an interview schedule to ensure consistency of discussion across all interviews, and was piloted prior to data collection to increase reliability (Supplement S2).

The interview began with the interviewer introducing themselves and their background and detailed the purpose of the conversation, which was to discuss physical activity, sedentary behaviour, and sleep, and that these were all the activities the individual might take part in during a 24 hour day. Adolescents were informed there were no right or wrong answers and to let the researcher know if they would like to stop or had any issues throughout the discussion. Adolescents were asked an ‘ice breaker’ question prior to the main discussion to allow the adolescent to relax and become comfortable with the discussion [25].

Interview questions were open-ended and prompts were utilised throughout to obtain greater detail and depth of discussion [26]. Data saturation (i.e., additional data no longer yielded new insights, themes, or information) was obtained at 15 participants, and recruitment stopped [27]. Data saturation was confirmed when no new themes emerged in the final two interviews and was verified through weekly debriefs with the research team, where the interview content and coding progress were collaboratively discussed. This is consistent with current research, suggesting that saturation is typically achieved within 9–17 interviews in studies with homogenous populations and focused research aims [28].

### 2.4. Data Analysis

The recordings of the interviews were transcribed using intelligent verbatim due to the volume of utterances and repetitions present within this age group [29]. Additionally, this ensured that no unethical stigmatisation of this group was conveyed [30]. Transcription was completed by the researcher who conducted the interview to ensure familiarity of the data. Throughout the transcription process, potential themes and reoccurring concepts were noted. Participant names were removed from the transcripts and replaced with their participant ID to ensure anonymity. Transcribed interviews were transferred to NVivo version 20.7.1 (Qualitative Solutions and Research International) to facilitate the organisation of codes, themes, subthemes, and relevant quotations.

There were four researchers involved in the coding of data. Interview data was primarily analysed by the lead researcher (M.P.), with continuous revision and input (A.K., X.J., and M.C.) using thematic analysis [31]. Thematic analysis is an iterative process moving between six phases to identify and analyse patterns within data. The six phases involve (1) familiarisation of the data (transcribing, reading, and annotating interview transcripts); (2) generating initial codes (listing initial interesting ideas and organising these into meaningful groups); (3) searching for themes (sorting codes into subthemes); (4) reviewing themes (refining themes, ensuring codes within themes are cohesive, and ensuring distinctions between themes); (5) defining and naming themes; and (6) producing the report (themes written and demonstrated through quotations). The defining of codes was mainly inductive; however, the lead researcher had a theoretical understanding of potential topics that may have arisen in the data, so the definition of codes could also be deductive.

### 2.5. Researcher Reflexivity

The lead researcher acknowledged and addressed their own biases, preconceptions, and personal experiences that could impact data collection and interpretation through reflexivity prior to the interviews (e.g., reflection on own biases, beliefs, and values), post interview (e.g., interview and participant impressions), and during the analysis of data (e.g., rationale for themes/codes and initial impressions of data) [31,32].

For example, the researcher acknowledged a belief in the value of movement behaviours for health as well as the potential power dynamic between the researcher and participant, and understood that they had no lived experience of movement behaviours in the context of type 1 diabetes that may have influenced the question-phrasing or the emphasis placed on certain topics. To mitigate this, a semi-structured interview guide was used. Additionally, a brief statement was developed to remind the researchers of their position throughout the research stages (“I value movement behaviours and recognise my position as an adult researcher without lived experience of type 1 diabetes. I hold awareness of my power and perspective and will listen without assumption”).

## 3. Results

### 3.1. Participant Demographics

In total, 15 adolescents (6 male; 9 female) participated, averaging 14.6 ± 2.0 years in age (8 younger adolescents; 7 older adolescents), with the majority identifying as white British (*n* = 14). Participants had a diabetes duration of 3.7 ± 3.1 years; an HbA_1c_ of 57 mmol/mol (7.4 ± 1.0%), which is within the recommended target values for HbA_1c_ in this age group; and a mean PedsQL 3.2 total score of 62.7 ± 14.3, indicating good quality of life. The sample predominantly used continuous glucose monitors (CGMs) (80%) and insulin pumps (67%) and, according to the accelerometer data collected, were sleeping 8.1 ± 0.7 h·per day, performing 28.1 ± 24 min·per day of MVPA, and had 9.8 ± 1.7 h·per day of sedentary time (Table 1).

**Figure 1:**
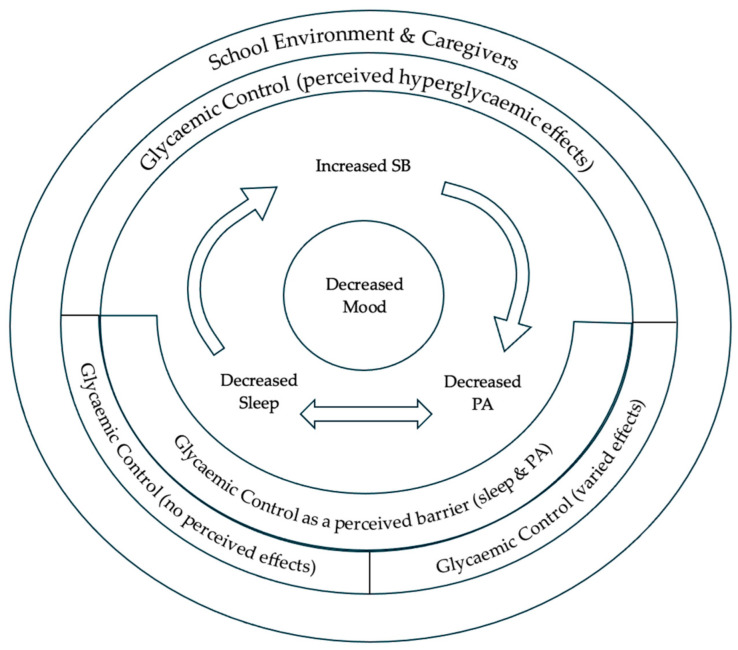
Conceptual representation of adolescents’ perceptions of 24-hour movement behaviours within the context of type 1 diabetes. At the centre, adolescents understood a cycle between sleep, physical activity (PA), and sedentary behaviour (SB), where increased SB led to a decrease in PA and, ultimately, decreased sleep. Importantly, adolescents acknowledged that this cycle had negative effects on mood. Adolescents also understood a bi-directional relationship between PA and sleep (indicated by the double arrow). Adolescents frequently discussed glycaemic control as a barrier to sleep and PA. Conversely, adolescents perceived each behaviour to have differing effects on glycaemic control. Adolescents perceived SB to have hyperglycaemic effects, PA was perceived to have varied effects on glycaemic control, and sleep had no perceived effects on glycaemic control. School environment and caregivers were frequently discussed by adolescents as contextual forces shaping movement behaviours and subsequent outcomes.

There were four major themes and five subthemes identified through the investigation into the perspectives and values towards movement behaviours, collectively, of adolescents with type 1 diabetes (Table 2). These are presented and supported with quotes from the adolescent. Figure 1 was created to conceptualise adolescents’ perceptions of 24-h MBs within the context of type 1 diabetes.

### 3.2. Theme 1: Sleep and Physical Activity Understood and Valued Above Sedentary Behaviour

When asked about all three movement behaviours, it was clear that sleep and physical activity were considered in adolescent lives, whereas sedentary behaviour was not consciously considered. One adolescent discussed sleep and physical activity and how these behaviours integrated into their life but needed reminding of sedentary behaviour. Even when adolescents were prompted, they struggled to discuss sedentary behaviour, with one adolescent explaining that they did not consider it within their day. Adolescents were aware of sleep and physical activity guidelines but had less awareness of sedentary behaviour guidelines. The prioritisation of sleep and physical activity was further highlighted through adolescents discussing how they tracked the two behaviours using their own wearable devices but omitted discussion of sedentary behaviour tracking. One adolescent expressed surprise that sedentary behaviour was tracked by their own wearable device, which was only realised during the interview.

*“Physical activity, well it depends how old you are, but I think it’s an hour a day or something like that on average and then seven to eight hours of sleep, honestly, I have no idea about sitting.”*

(P12, female, and 16 years of age).

*“The only thing I really track is how many steps I’ve done and how much sleep I get.”*

(P6, male, and 14 years of age).

*“I think I can get a bit obsessive with things that show you so exactly like an Apple Watch does. I noticed that I was doing that—oh it had stand hours on it! Sorry I just realised that for the sitting thing.”*

(P3, female, and 18 years of age).

### 3.3. Theme 2: Recognition of Movement Behaviours’ Interconnection

Throughout the discussion, adolescents exhibited an underlying awareness of the relative or time-dependent principles underpinning a 24-h MB approach. They expressed understanding that a change in one behaviour can have a significant impact on another. Adolescents understood the interconnectedness of all three movement behaviours and discussed a “cycle” between sleep, physical activity, and sedentary behaviour. They believed that their participation in sedentary behaviour was at the expense of their physical activity, ultimately resulting in adolescents experiencing poorer sleep. Adolescents also described how poor sleep resulted in physical activity being replaced with increased time spent sedentary. Adolescents understood how participation in one behaviour might affect another, highlighting the understanding of a bi-directional relationship between physical activity and sleep. Additionally, adolescents described how mood mediated the influence of good sleep on physical activity as well as the interaction between sedentary behaviour and physical activity. However, adolescents also perceived sedentary behaviour as relaxing and as a behaviour that would replenish them after physical activity participation.

*“I am literally sitting at my desk all day working and I don’t have time to do any kind of exercise. Then also that affects my sleep as well because I might be mentally tired, but I am not like physically tired because I have not done any physical activity which makes it a bit more difficult to get to sleep.”*

(P1, female, and 16 years of age).

*“If you haven’t slept a lot then the next day you don’t want to be going out for loads of walks, you don’t want to go out for a run, you don’t want to ride a bike you just kind of sit and you just kind of do nothing.”*

(P11, female, and 17 years of age).

*“If you have a more restful sleep and like a good sleep you will be more energetic so you will have more energy to do exercise.”*

(P10, male, and 16 years of age).

*“If I do a physical activity then I would sit down after just because I’m tired.”*

(P6, male, and 14 years of age).

### 3.4. Theme 3: Movement Behaviours’ Interaction with Health Outcomes

#### 3.4.1. Mood

Adolescents discussed how they perceived movement behaviours to interact with their mood. They spoke about poorer sleep and higher sedentary behaviour producing negative moods while higher levels of physical activity produced positive moods. When adolescents discussed all three behaviours together, they discussed the combination of poor sleep, higher sedentary behaviour, and lower physical activity being detrimental to mood.

*“I think if I’m sat down for long periods of time or even if it were short periods of time broken up for just a long day it just makes you feel a bit… you know. Yeah, and then, that can kind of effect your mood.”*

(P12, female, and 16 years of age).

*“You can’t just say ‘I’m going to sit down and do nothing’ you say, ‘I am going to go do exercise because it’s good for me’, it’s good for my physical and mental health.”*

(P14, male, and 13 years of age).

*“If I have a day and I have been sitting around all day, I’ve not done any exercise and got no sleep I get really grumpy.”*

(P5, female, and 13 years of age).

#### 3.4.2. Glycaemic Control

There were mixed perceptions from adolescents on how movement behaviours interacted with glycaemic control. Physical activities’ interaction with glycaemic control was discussed in relation to different intensities of the behaviour. Adolescents recognised the short-term varying effects of different intensities of physical activity on glycaemic control. However, the effects of different physical activity intensities were confused at times. Adolescents discussed the hypoglycaemic effects of light physical activity but also highlighted light physical activity being used as a blood glucose management tool to bypass potential hyperglycaemic events. Adolescents highlighted that they were more likely to go hyperglycaemic if they had higher sedentary levels. Additionally, adolescents did not perceive better sleep to have direct effects on glycaemic control. Instead, adolescents mainly discussed sleep in relation to sleep interruptions due to glucose management requirements through the night.

*“I’ve forgotten which way around it is but anaerobic and aerobic exercises. One will make your blood sugars go up and one of them will make you go down straight away.”*

(P14, male, and 13 years of age).

*“If you’re sitting, you might need to get up because if you’ve been sitting for too long your blood might go a little bit high.”*

(P4, male, and 14 years of age).

*“I think the thing with sleep is it doesn’t affect my blood sugars so the issue in sleep is if something happened that has made my blood sugar go low whilst I’m asleep.”*

(P1, female, and 16 years of age).

#### 3.4.3. Glycaemic Control as a Barrier to Movement Behaviours

Glycaemic control was also discussed by adolescents as a barrier to movement behaviours, particularly sleep, where the discussion of sleep/night-time interruptions from diabetes technology (e.g., CGMs and pumps) was prevalent. Glycaemic control was a perceived barrier to physical activity, with many adolescents discussing the importance of ensuring that blood glucose was at an optimal level before physical activity could be performed.

*“I am sleeping with an actual machine attached to me. It’s more difficult, I think. I know I’ve had sleep issues since before I was diagnosed but notable ones since I was diagnosed.”*

(P3, female, and 18 years of age).

*“When I was on my way home from school I was like ‘oh it would be really nice to do a workout today’ but I don’t think I will be able to because I don’t think I will be able to get my blood sugar to go up for long enough. That makes me feel annoyed because it’s inconvenient. If that was someone without diabetes that wouldn’t be a problem.”*

(P1, female, and 16 years of age).

### 3.5. Theme 4: Movement Behaviours Within the Environmental Context of the Adolescent

#### 3.5.1. School

Throughout the discussions, adolescents highlighted how the school environment promoted physical activity, broke up sedentary behaviour, and facilitated better sleep. Although adolescents discussed how the school environment created opportunities to be active, often in relation to physical education, many discussed the barriers of performing physical activity within a school setting due to the resulting type 1 diabetes management requirements, which in turn created negative affective states such as anxiety and stress. Adolescents acknowledged school provided a structure that could break up long periods of sedentary behaviour and provide a good sleep routine. Another adolescent described how the school day provided a good structure to balance movement behaviours specifically for type 1 diabetes management. However, adolescents discussed how the school environment might promote an unhealthier balance of behaviours, such as schoolwork and exams promoting higher levels of sedentary behaviour and the challenges of early school start times, which were often intensified by the night-time awakenings caused by diabetes alarms.

*“Physical activity, I probably should be doing more of it to be honest because I only really do it in school and maybe a bit at home.”*

(P15, female, and 12 years of age).

*“I usually go to my bed at like half nine or ten now because I’m in a school routine but when I’m not it’s kind of all over the place and my sleep pattern is just so all over the place. It kind of gets ruined and I feel icky.”*

(P9, female, and 16 years of age).

*“So, at school I will find that it’s kind of a decent environment because I will be sitting down for a couple of lessons for a few hours and then I go out with my friends for a walk which is a mile or so then come back. So, I find they balance each other fairly nicely.”*

(P11, female, and 17 years of age).

*“I think just the social pressure of knowing that if my blood sugars were good and I went and did some form of physical activity, if I had a hypo in the middle of a gym hall that was embarrassing for me.”*

(P3, female, and 18 years of age).

*“Sitting, I guess I do it a lot because I’ve got a lot of homework and then studying.”*

(P2, female, and 13 years of age).

*“So, say I hadn’t a decent nights sleep especially if my alarm had been going off or I didn’t go to sleep till late and sometimes I have to wake up early for school. I will be tired and then I might be quite moody and then because I’m tired my levels might go high which then stresses me out because I will be at school, and I don’t like injecting in public. So, then I might get more stressed, and it might make me quite anxious.”*

(P11, female, and 17 years of age).

#### 3.5.2. Caregivers

When adolescents discussed their movement behaviour participation when they were younger, it was clear they believed that they had less autonomy in how they engaged in behaviours. Instead, adolescents highlighted the importance of their caregivers in engaging in these behaviours and recognised how they had more autonomy as they aged. Additionally, when adolescents were asked who they would ask for any information related to movement behaviours, they highlighted their caregivers. Finally, adolescents discussed how caregivers’ own movement behaviours were impacted, particularly sleep, as they often woke to monitor their child’s glucose levels throughout the night.

*“When I was eleven, I would probably get more sleep because I would go to bed earlier, and my parents would expect me to go to bed earlier. But then as I get older, I go to sleep whenever I want, and you have potentially less sleep and also end up just getting less sleep to get up early to go to school and work.”*

(P1, female, 16 years of age).

“I’m a very deep sleeper so, if I’ve got a high I just sleep like this *imitates sleeping* and my dad just like comes in and says ‘[NAME]’ and I’m like half-awake. So, he just types it into the pump anyway, so it is not an issue.”

(P14, male, 13 years of age).

## 4. Discussion

This is the first study, to our knowledge, investigating the perceptions of adolescents living with type 1 diabetes towards all three movement behaviours together and how they might interact within a day. We highlighted four main themes of adolescents’ perceptions towards these behaviours. Adolescents discussed the interconnected nature of all behaviours within a day, the importance of sleep and physical activity with a lower consideration of sedentary behaviour, the interaction that movement behaviours have with health outcomes, and movement behaviours’ interaction with their environment.

Although previous research has consistently highlighted the benefits of reduced sedentary behaviour for adolescents with [5] and without type 1 diabetes, it appears that the importance of the behaviour has not been adequately conveyed to this population [33]. Within type 1 diabetes clinical care, sedentary behaviour accompanies physical activity and its importance is somewhat overshadowed. Current international physical activity clinical guidelines for adolescents with type 1 diabetes state ‘Children and adolescents should limit the amount of time spent being sedentary, particularly the amount of recreational screen time’, with no other sedentary-behaviour-specific recommendations [6]. If the communication of sedentary behaviours’ importance has not already been translated to adolescents with type 1 diabetes, then perhaps a 24-h MB approach highlighting the importance of a healthy balance of each behaviour will reframe sedentary behaviours’ importance and facilitate optimal health outcomes.

Adolescents frequently discussed how a balance of behaviours aligning with 24-h MB recommendations could benefit their mental health, which is consistent with previous research in adolescents [34]. Specifically, discussions surrounding how all three behaviours impacted motivation and fatigue were prevalent. Motivation and fatigue can have a profound impact on the ability of a person to exert effort or complete tasks [35], which is of great significance to adolescents with type 1 diabetes as they are consistently required to conduct daily self-management activities. Although research has previously highlighted the negative impact that poor sleep can have on self-management activities [36], our research suggests physical activity and sedentary behaviour should also be considered alongside sleep to optimise self-management activities and mental health.

Throughout discussions, the caregivers and school environment were consistently referred to in relation to adolescents’ movement behaviours. Adolescents discussed how their caregivers’ behaviour, specifically in relation to sleep, was often influenced by their own type 1 diabetes management requirements (e.g., sleep disruptions due to alarms) [37]. Considering the crucial role of caregivers in supporting adolescents living with type 1 diabetes with their everyday management decisions and behaviours, particularly younger adolescents with lower autonomy, it is important that interventions aimed at promoting a healthier balance of movement behaviours also consider the caregivers’ behaviours [38]. Research is beginning to investigate interventions aimed at promoting healthier movement behaviours in schools, using activities to improve movement behaviour knowledge [39,40]. Such an intervention might be adapted to cater to adolescents with type 1 diabetes, with a focus on information promoting the importance of reduced sedentary behaviour, assigning active homework and limiting the amount of homework assigned [41] and flexibility in physical education (PE) involvement, including relevant training for PE staff, the presence of type 1 diabetes emergency first aid kits, and the availability of type 1 diabetes ‘safe spaces’ to complete management activities [42].

Although there are many strengths of this research, including a focus on the adolescents’ perspective, facilitation of strong interviewer–participant rapport, and rigorous thematic analysis conducted by trained researchers, there are some limitations. Firstly, the small sample size (*n* = 15) and predominantly white British participants limit the generalisability of the findings. The homogeneity of the sample may not reflect the diverse experiences of adolescents from different ethnic and socioeconomic backgrounds. Additionally, most participants had access to advanced diabetes management technologies, which could influence their perceptions of movement behaviours and may not be representative of the broader type 1 diabetes adolescent population. Secondly, the participants’ prior movement behaviour habits might have shaped their perceptions and ability to reflect on physical activity, sleep, and sedentary behaviour. Given that participants had recently completed a study involving accelerometer-based movement behaviour measurements, they may have been more attuned to certain behaviours. Finally, the study involved interviews exclusively with adolescents. Younger adolescents might struggle to articulate their thoughts, potentially impacting the depth of the data. Future research should consider larger, more diverse samples and include perspectives from caregivers and healthcare providers to enhance the robustness and applicability of the findings.

## 5. Conclusions

This study highlights the awareness of adolescents with type 1 diabetes regarding the interconnectedness of movement behaviours and the positive influence a balanced approach can have on physical and mental health. The findings provide important information for future interventions to promote holistic movement behaviour interventions for type 1 diabetes management that target the adolescent, their school environment, and their caregivers.

## Figures and Tables

**Table 1:** Participant characteristics for the full sample. Frequency data is given as number (%); descriptive data is given as mean ± standard deviation (M; SD).

Characteristic	Full Sample (*n* = 15)
Interview Length (Minutes)	23 ± 6.5
Age	
Younger Adolescent (11–15 Years)	8 (53.3)
Older Adolescent (16–18 Years)	7 (46.7)
Sex	
Female	9 (60)
Male	6 (40)
Ethnicity	
White	15 (100)
Insulin Delivery	
Pen	5 (33.3)
Pump	10 (66.7)
Blood Glucose Measurement Method	
CGM	12 (80)
Finger-Prick	3 (20)
Age (Years)	14.6 ± 2.0
Diabetes Duration (Years)	3.7 ± 3.1
HbA_1c_ (%)	7.4 ± 1.0
Paediatric Quality of Life Total Score	62.7 ± 14.3
Sleep (Hours·per Day) ^a^	8.1 ± 0.7
SED (Hours·per Day) ^a^	9.8 ± 1.7
MVPA (Min·per Day) ^a^	28.1 ± 24

HbA_1c_: haemoglobin A1c; CGM: continuous glucose monitor; SED: sedentary behaviour; MVPA: moderate-to-vigorous physical activity. ^a^ 24-hour movement behaviour data, using actigraphy available from 12 participants.

**Table 2:** Codebook of themes.

Theme	Subtheme	Definition
Theme 1: Sleep and PA understood and valued above SB		Adolescent understands and values sleep and PA more than SB
Theme 2: Recognition of movement behaviours’ interconnection		Adolescent perceptions on how sleep, PA, and SB might interact and impact one another
Theme 3: Movement behaviours interaction with health outcomes	Mood	Adolescent perceptions on how sleep, PA, and SB interact with their mood
	Glycaemic control	Adolescent mixed perceptions on how sleep, PA, and SB interact with their glycaemic control
	Glycaemic control as a barrier to movement behaviours	Adolescent perceptions of glycaemic control as a barrier to movement behaviours
Theme 4: Movement behaviours within the environmental context of the adolescent	School	Adolescent perceptions of school and how it affects their sleep, PA, and SB participation and understanding
	Caregivers	Adolescent perceptions of their caregivers’ role in their sleep, PA, and SB

PA: physical activity; SB: sedentary behaviour.

## Data Availability

Data is available upon request to the corresponding author.

## Supplementary Materials

The following supporting information can be downloaded at https://www.mdpi.com/article/10.3390/ijerph22081295/s1, Supplement S1: Accelerometer data processing information and rationale [43,44,45,46,47,48,49,50]; Supplement S2: Adolescent semi-structured interview guide.

## Abbreviations

The following abbreviations are used in this manuscript:

PA	Physical Activity
MVPA	Moderate-to-Vigorous Physical Activity
SB	Sedentary Behaviour
24-h MB	24-Hour Movement Behaviour
T1D	Type 1 Diabetes
