# Supplementary material for: “If You Haven’t Slept a Lot (…) You Don’t Want to Go Out for a Run, You Don’t Want to Ride a Bike, You Just Kind of Sit and You Just (…) Do Nothing”—Perceptions of 24-Hour Movement Behaviours Among Adolescents Living with Type 1 Diabetes
Source: Int J Environ Res Public Health. 2025 Aug 19;22(8):1295. doi: 10.3390/ijerph22081295 (PMC12386231; doi:10.3390/ijerph22081295)
Supplement: Supplementary file 1 [file ijerph-22-01295-s001.zip › Supplementary S1.pdf]

Supplement S1: Accelerometer Data Processing Information and Rationale

Accelerometer Considerations	Decision	Rationale
Wear location	Non-dominant wrist (Fairclough et al., 2016)	Improved compliance, improved data capture of all 24-h MB, no evidence to suggest dominant or non-dominant wrist is more accurate
Activity cut-points	*Hildebrand (2014, 2017)* cut points based on the ENMO (mg per 1s).	Frequently utilised in 24-h MB adolescent studies, aligns with the research shift in utilising raw device-agnostic data.
Sleep algorithms	*van Hees (2015)* heuristic sleep algorithm based on changes in the arm (angle z)	Sleep-wake detection without sleep diaries aids compliance issues in adolescents, aligns with the research shift in utilising raw device-agnostic data.
Non-wear time	*van Hees (2013)* non-wear algorithm based on the standard deviation and range of each axis.	Frequently utilised in 24-h MB adolescent studies, outperforms epoch-based algorithm to detect non-wear time, aligns with the research shift in utilising raw device-agnostic data.
Valid day	≥10 hours/day (≥600 minutes/day)	Frequently utilised in 24-h MB adolescent studies.
Valid week	≥4 days valid week including ≥3 valid weekdays and ≥1 valid weekend day (Fairclough et al., 2023)	More representative of typical weekly behaviours which can

		vary substantially on weekdays and weekends.
Sampling frequency	80Hz	High frequency that also accommodates for device battery life for 2-weeks of 24-hour wear.
Epoch length	Not Applicable	Not strictly required for raw acceleration metrics such as ENMO; a 1-second epoch is commonly applied to align with Hildebrand cut-points.
Axis analysed	Three	Required to compute the ENMO metric $(x^2 + y^2 + z^2)^{½} – 1)$

References:

Duncan S, Stewart T, Mackay L, Neville J, Narayanan A, Walker C, et al. Wear-Time Compliance with a dual-accelerometer system for capturing 24-h behavioural profiles in children and adults. Int J Environ Res Pub Health. 2018;15(7):1296.

Fairclough SJ, Noonan R, Rowlands AV, Van Hees V, Knowles Z, Boddy LM. Wear compliance and activity in children wearing wrist- and hip-mounted accelerometers. *Med Sci Sports Exerc.* (2016) 48:245–53. doi: 10.1249/MSS.0000000000000771

Fairclough, S. J., Rowlands, A. V., del Pozo Cruz, B., Crotti, M., Foweather, L., Graves, L. E., ... & Boddy, L. M. (2023). Reference values for wrist-worn accelerometer physical activity metrics in England children and adolescents. *International Journal of Behavioral Nutrition and Physical Activity*, *20*(1), 35.

Supplement S1: Accelerometer Data Processing Information and Rationale

Hildebrand M, VAN Hees VT, Hansen BH, Ekelund U. Age group comparability of raw accelerometer output from wrist- and hip-worn monitors. *Med Sci Sports Exerc.* (2014) 46:1816–24. doi: 10.1249/MSS.0000000000000289

Hildebrand, M., Hanson, B.H., van Hees, V.T., & Ekelund, U. (2017). Evaluation of raw acceleration sedentary thrsholds in children and adults. *Scandinavian journal of medicine & science in sports, 27(12), 1814-1823.*

Rosenberger ME, Fulton JE, Buman MP, Troiano RP, Grandner MA, Buchner DM, et al. The 24-hour activity cycle: a new paradigm for physical activity. Med Sci Sports Exerc. 2019;51(3):454–64.

van Hees VT, Sabia S, Anderson KN, Denton SJ, Oliver J, Catt M, et al. A novel, open access method to assess sleep duration using a wrist-worn accelerometer. PLoS One. 2015 Nov 16;10(11):e0142533.

van Hees, V. T., Gorzelniak, L., Dean León, E. C., Eder, M., Pias, M., Taherian, S., . . . Horsch, A. (2013). Separating movement and gravity components in an acceleration signal and implications for the assessment of human daily physical activity. *PLoS One, 8*(4), e61691.
