# Supplementary material for: “If You Haven’t Slept a Lot (…) You Don’t Want to Go Out for a Run, You Don’t Want to Ride a Bike, You Just Kind of Sit and You Just (…) Do Nothing”—Perceptions of 24-Hour Movement Behaviours Among Adolescents Living with Type 1 Diabetes
Source: Int J Environ Res Public Health. 2025 Aug 19;22(8):1295. doi: 10.3390/ijerph22081295 (PMC12386231; doi:10.3390/ijerph22081295)
Supplement: Supplementary file 1 [file ijerph-22-01295-s001.zip › Supplementary S2.pdf]

Welcome and Topic Overview

Hello and welcome to the session. [INTERVIEWER INTRODUCES THEMSELVES].

We will be talking about specific activities like physical activity, sitting and sleep. These are all the activities you might take part in during the 24-hour day.

Ground Rules

Before we start having a chat, I just want to say that there are no right or wrong answers to the questions I ask, I am interested in hearing everything and anything you have to say.

You can talk about good things and bad things and your honest opinion to the questions I ask you would be great! All your comments will be useful, and I look forward to hearing them.

During our chat, we will be on a first name basis but don't worry we will not use any names in our report. We will also remove any information you mention during the interview that could identify you. You can be assured that no information will be shared outside this discussion to anyone unless I feel like you might need some additional help (for example, if you get really upset chatting about some things you are asked).

I will be audio and video recording these interviews as I don't want to miss any of your useful comments. I would like to be completely present and listen carefully to everything you say, so having audio and video will help me analyse the data later. I would just like to confirm the audio and video recording of this interview is ok with you before we begin?

Opening Question

Well, lets begin. Hopefully you can see my name on the screen as a reminder, but you pronounce my name as BLANK. Please just let me know if you need anything throughout the interview.

Main Questions

Supplement S2: Adolescent Semi-structured Interview Guide

Ok, if you are ready to start, we can start chatting about the activities you might do in a day remember these include physical activity, sitting and sleep.

Awareness

1. What do you think about each of these activities?

2. Are you aware of any activity recommendations or advice?

    a. Is there somewhere specific you get these recommendation/advice?

Impact

3. How would you describe a day where you had good activity patterns?

    a. How would this affect your mood and glucose control?

4. How would you describe a day where you had bad activity patterns?

    a. How would this affect your mood and glucose control?

5. Who would you say is most affected by your own activity patterns?

    a. Why did you choose this individual?

Evolution

6. How do you believe your activity patterns change as you grow up?

    a. Why do you think this is?

Closing Questions

1. Would you like to add any other information related to our discussion today?

2. Are there any other components that would be of importance to discuss that we have not touched on today?
3. Do you have any comments or questions for me?

Once all comments have been received by participants:

Thank you very much for your participation. If you have any questions, then please do not hesitate to contact me.
